# Squeezing effects applied in nonclassical superposition states for quantum nanoelectronic circuits

**DOI:** 10.1186/s40580-017-0111-4

**Published:** 2017-06-29

**Authors:** Jeong Ryeol Choi

**Affiliations:** Department of Radiologic Technology, Daegu Health College, Yeongsong-ro 15, Buk-gu, Daegu, 41453 Republic of Korea

## Abstract

Quantum characteristics of a driven series RLC nanoelectronic circuit whose capacitance varies with time are studied using an invariant operator method together with a unitary transformation approach. In particular, squeezing effects and nonclassical properties of a superposition state composed of two displaced squeezed number states of equal amplitude, but 180° out of phase, are investigated in detail. We applied our developments to a solvable specific case obtained from a suitable choice of time-dependent parameters. The pattern of mechanical oscillation of the amount of charges stored in the capacitor, which are initially displaced, has exhibited more or less distortion due to the influence of the time-varying parameters of the system. We have analyzed squeezing effects of the system from diverse different angles and such effects are illustrated for better understanding. It has been confirmed that the degree of squeezing is not constant, but varies with time depending on specific situations. We have found that quantum interference occurs whenever the two components of the superposition meet together during the time evolution of the probability density. This outcome signifies the appearance of nonclassical features of the system. Nonclassicality of dynamical systems can be a potential resource necessary for realizing quantum information technique. Indeed, such nonclassical features of superposition states are expected to play a key role in upcoming information science which has attracted renewed attention recently.

## Background

Present high technologies of lithography and crystal growth paved the way for sophisticated experiments [1, 2] with nano materials, leading to the rapid development of nanoscale technology in the field of nano physics and nanoelectronics. It is well known from diverse theories and elegant experiments related to nano materials that the quantum effects are conspicuous for nano devices, especially when the transport dimension reaches a threshold value which is so-called the Fermi wavelength. Consequently, the understanding of quantum properties which appear in such nanoscale materials is important, meanwhile classical description for the motion of charge carriers is no longer valid in that situation. This is the reason why the research for the underlying theory of fundamental quantum characteristics of nano devices and nano electronic circuits are crucial for the development of future science and technology which will inevitably be associated with nano dimension.

For this reason, quantum features of nanoelectronic circuits have been extensively investigated in the literature so far [3–8]. Although motion of charges in fundamental LC circuits is described by a simple Hamiltonian that does not vary with time, a large category of nanoelectronic circuits, such as LC circuits driven by a sinusoidal power source and series RLC circuits that have time-varying parameters is classified as a time-varying system [9–11] that can be described in terms of a time-dependent Hamiltonian. In this work, we focus on the study of a nanoelectronic circuit involving a time-varying capacitance and a power source [12–20]. The mathematical treatment of time-dependent Hamiltonians requires special techniques, such as an invariant operator method, a canonical or unitary transformation approach, a reduction method, a propagator method, and some others as well [21–24].

Quantum properties of a series RLC nanoelectronic circuit that involves a time-dependent driving power source will be investigated in this work by adopting an invariant operator method together with a unitary transformation approach, that is very useful for managing a system characterized by time-dependent parameters. The invariant operator method was firstly introduced by Lewis and Riesenfeld in time-dependent harmonic oscillator systems [21]. The merit of this method in the quantum domain is that the eigenstates of an invariant operator are closely related with the Schrödinger solutions of the system. In fact, we can obtain exact quantum solutions by multiplying eigenstates of the invariant operator by suitable phase factors. This implies that it is required, for studying quantum properties of the system, to derive the eigenstates of the invariant operator by solving its eigenvalue equation. However, the eigenvalue equation may not be easily solved since the system we regard is somewhat complicated due to the existence of time-varying parameters. In order to overcome this difficulty, a unitary transformation approach will be considered. By introducing an appropriate unitary operator, it is possible to transform the original invariant operator to a simple form whose eigenstates are well known or easily derivable. From the inverse transformation of the eigenstates associated to the transformed system, one can evaluate full eigenstates in the original system. Finally, by determining the quantum phase factors via the aid of the Schrödinger equation, the complete quantum solutions in the number state can be obtained.

On the basis of quantum solutions derived in this way, we will study displaced squeezed number states (DSNSs) [25]. While displaced number states (DNSs) [20, 26, 27] can be obtained by operating only the displacement operator on the wave functions in number states, DSNSs are obtained by first operating the squeezing operator, and then the displacement operator on the same states. DSNSs are known as one of the familiar nonclassical states that can be analyzed in terms of the Q-parameter proposed by Mandel. Moreover, DSNSs reveal the properties of sub-Poissonian or super-Poissonian statistics depending on the relative scale of parameters that endow a particular realization of states. For more details for this, refer to Ref. [28].

The main goal of this work is to investigate squeezing effects and nonclassical features of superposition states of quantum nanoelectronic circuits, considering the case that the elements of the superposition states are composed of two DSNSs with equal amplitudes but 180° out of phase. The superposition of two DSNSs with an opposite or arbitrary phase difference exhibits interference that is highly nonclassical, as well as some properties similar to those of statistical mixtures. Such states are known as a family of Schrödinger-cat states which can be used as potential resources for qubits in superconducting circuits that process quantum information [29, 30]. El-Orany and Obada studied the effects of quantum mechanical interference between two individual DSNSs with 180° out of phase and addressed nonclassicality of the system such as negativity of the Wigner function, quadrature squeezing, purity, etc. [31]. A method for generating superposition states composed of DSNSs with high fidelity is recently proposed by Podoshvedov [32]. We will derive exact wave functions of superposition states composed of DSNSs. The time evolution of these quantum states will be studied in detail. The effects of time dependence of parameters on the expectation value of canonical variables will also be investigated under the choice of particular time-variable parameters.

## Results and discussion

### Hamiltonian dynamics

A series RLC nanoelectronic circuit driven by an arbitrary power source is considered in this work. We assume that the capacitance of the system varies with time. If we denote the charge stored in the capacitor as *q*, we obtain a differential equation from Kirchhoff’s law as

1 $$\begin{aligned} \ddot{q} + \frac{R}{L} \dot{q} + \omega ^2(t) q = \frac{\mathcal {E} (t)}{L}, \end{aligned}$$

where $\omega (t) = [LC(t)]^{-1/2}$ and $\mathcal {E} (t)$ is the driving power source. We can say that the complete classical solution of this equation is represented as

2 $$\begin{aligned} Q(t)=Q_{c}(t) +Q_p(t), \end{aligned}$$

where $Q_{c}(t)$ is a complementary function and $Q_p(t)$ is a particular solution. We can do the same thing for the conjugate canonical current, such that

3 $$\begin{aligned} P(t)=P_{c}(t) +P_p(t). \end{aligned}$$

Once the solution of $Q_{c}(t)$ and $Q_p(t)$ are known, we can also have $P_{c}(t)$ and $P_p(t)$ from

4 $$\begin{aligned} P_{c}(t)=L e^{(R/L)t} d {Q}_c (t)/dt, \end{aligned}$$

5 $$\begin{aligned} P_{p}(t)= L e^{(R/L)t} d {Q}_p (t)/dt. \end{aligned}$$

The Hamiltonian that yields the classical equation of motion given in Eq. (1) can be written as

6 $$\begin{aligned} {\hat{H}} = e^{-(R/L)t} \frac{{\hat{p}}^2}{2L} + \frac{1}{2}e^{(R/L)t} \left[ \omega ^2(t)L {\hat{q}}^2 - 2 \mathcal {E} (t) {\hat{q}} \right] , \end{aligned}$$

where ${\hat{p}} = -i\hbar \partial / \partial q$, which stands for the operator of the canonical current.

We introduce an annihilation operator of the form

7 $$\begin{aligned} {\hat{A}} = \sqrt{\frac{1}{2\hbar L \omega _0}} \left\{ \left[ \frac{\rho _0 \omega _0}{\rho (t)} - i e^{(R/L)t} \frac{\dot{\rho }(t)}{\rho _0} \right] L [{\hat{q}}- Q_p(t)] + i \frac{\rho (t)}{\rho _0} [{\hat{p}}- P_p(t)] \right\} , \end{aligned}$$

where $\omega _0 = \omega (0)$, $\rho _0$ is an arbitrary real constant, and $\rho (t)$ is a solution of the following differential equation

8 $$\begin{aligned} \ddot{\rho } (t) + \frac{R}{L} \dot{\rho } (t)+ \omega ^2(t) \rho (t) - e^{-2(R/L)t} \frac{(\omega _0\rho _0^2)^2}{\rho ^3(t)} =0 . \end{aligned}$$

Of course, the hermitian conjugate of Eq. (7), ${\hat{A}}$ ^†^, is the creation operator. Notice that ${\hat{A}}$ and ${\hat{A}}$ ^†^ satisfy the boson commutation relation of the form [${\hat{A}}, {\hat{A}}$ ^†^] = 1. Now, we can establish an invariant operator in terms of ${\hat{A}}$ and ${\hat{A}}$ ^†^ such that [21]

9 $$\begin{aligned} {\hat{I}} = {\hbar {\omega }}_0 \left( {\hat{A}}^\dagger {\hat{A}} + \frac{1}{2} \right) . \end{aligned}$$

Due to the time dependence of parameters of the system, the Hamiltonian given in Eq. (6) is a somewhat complicated form. For this reason, it is favorable to simplify the problem. The time-dependent Hamiltonian can be transformed to a simple form by a suitable unitary operator ${\hat{U}}$. Let us consider the following unitary transformation of the Hamiltonian

10 $$\begin{aligned} {\hat{H}}' = {\hat{U}}^{-1} {\hat{H}} {\hat{U}} - i \hbar {\hat{U}}^{-1} \frac{\partial {\hat{U}}}{\partial t}, \end{aligned}$$

where a unitary operator ${\hat{U}}$ is chosen in the form [20]

11 $$\begin{aligned}{\hat{U}} &=\exp \left( \frac{iP_p(t) {\hat{q}} }{\hbar } \right) \exp \left( -\frac{iQ_p(t) {\hat{p}} }{\hbar }\right) \exp \left( \frac{i L \dot{\rho }(t) e^{(R/L) t} {\hat{q}}^2}{2\hbar \rho (t) }\right) \nonumber \\ & \quad \times \exp \left[ -\frac{i}{4\hbar } ({\hat{q}}{\hat{p}}+{\hat{p}}{\hat{q}}) \ln \left( \frac{\rho ^2(t)}{\rho _0^2} \right) \right] . \end{aligned}$$

Performing a straightforward algebra for Eq. (10) yields

12 $$\begin{aligned} {\hat{H}}{^\prime} = \frac{\rho _0^2 }{\rho ^2 (t)} e^{-(R/L)t} \left[ \hbar \omega _0 \left( {\hat{a}}^\dagger {\hat{a}} + \frac{1}{2} \right) \right] + \mathcal {L}_p (t), \end{aligned}$$

where

13 $$\begin{aligned}&{\hat{a}} = {\hat{U}}^{-1} {\hat{A}} {\hat{U}}, \end{aligned}$$

14 $$\begin{aligned}&{\hat{a}}^\dagger = {\hat{U}}^{-1} {\hat{A}}^\dagger {\hat{U}} , \end{aligned}$$

and $\mathcal {L}_p (t)$ is a time function of the form

15 $$\begin{aligned} \mathcal {L}_p (t) = e^{-(R/L)t} \frac{P_p^2(t)}{2L} - \frac{1}{2} e^{(R/L)t} \omega ^2(t)L Q_p^2(t). \end{aligned}$$

From a direct evaluation with Eqs. (13) and (14), we easily see that ${\hat{a}} = X{\hat{q}} + {iY{\hat{p}}}$ and ${\hat{a}}^\dagger = X{\hat{q}} - {iY{\hat{p}}}$, where $X=\sqrt{L\omega _0/(2\hbar )}$ and $Y=1/{\sqrt{2\hbar L \omega _0}}$. These correspond to the ladder operators of the simple harmonic oscillator with frequency $\omega _0$. We can also confirm that $[{\hat{a}},{\hat{a}}^\dagger ]=1$.

The equation for *q* in the transformed system is obtained by applying Hamiltonian dynamics with Eq. (12). Hence, from a minor calculation, the classical equation of motion in the transformed system is derived to be

16 $$\begin{aligned} \ddot{q} + \bigg ( \frac{R}{L} + 2\frac{\dot{\rho }(t)}{\rho (t)} \bigg ) \dot{q} + e^{-2(R/L)t}\frac{(\omega _0 \rho _0^2)^2}{\rho ^4(t)} q = 0 . \end{aligned}$$

Let us denote a classical solution (complementary functions) for this equation as $Q_{\mathrm{t},c}(t)$. Then the corresponding classical solution for conjugate canonical current is obtained from

17 $$\begin{aligned} P_{\mathrm{t},c}(t) = \frac{\rho ^2(t)}{\rho _0^2}L e^{(R/L)t} d {Q}_{\mathrm{t},c} (t)/dt . \end{aligned}$$

The quantities $Q_{\mathrm{t},c}(t)$ and $P_{\mathrm{t},c}(t)$ will be used for developing a quantum theory of the system in the subsequent sections.

### Superposition of displaced squeezed number states

The DSNS is defined by first squeezing the number state and, then, displacing it. The superposition of DSNSs as well as that of DNSs also exhibits many nonclassical characteristics, such as interference and phase fluctuations that can be applied to implementing quantum information techniques, while its generation requires high technology and novel ideas that have yet to be developed. As a strategy for investigating this state, we first derive the DSNS in the transformed system, and then, we transform it inversely in order to obtain the DSNS in the original system.

The squeeze operator in the transformed system is given by

18 $$\begin{aligned} {\hat{S}} (z) = \exp \left [-\frac{1}{2}(z^* {\hat{a}}^2 - z {\hat{a}}^{\dagger 2})\right], \end{aligned}$$

where *z* is a squeezing parameter that can be represented in terms of its magnitude *r* and phase $\phi$ such that

19 $$\begin{aligned} z = r e^{i \phi }. \end{aligned}$$

Using Eq. (65) in "Methods" (the last section), the squeeze operator can be rewritten as

20 $$\begin{aligned}{\hat{S}} (z) &= \frac{1}{\sqrt{s }} \exp \left[ \frac{i L \omega _0 }{2\hbar } \frac{\sin \phi \sinh r}{s} {\hat{q}}^2 \right] \exp \left[ -\frac{i}{\hbar } {\hat{q}} {\hat{p}}\ln s \right] \nonumber \\&\quad \times \exp \left [- \frac{i }{2 L \omega _0 \hbar } \frac{\sin \phi \sinh r }{s } {\hat{p}}^2 \right ], \end{aligned}$$

where

21 $$\begin{aligned} s= \cosh r + \cos \phi \sinh r . \end{aligned}$$

On the other hand, the displacement operator is defined to be

22 $$\begin{aligned} {\hat{D}} (\alpha ) = \exp (\alpha {\hat{a}}^\dagger - \alpha ^* {\hat{a}}) , \end{aligned}$$

where

23 $$\begin{aligned} \alpha = \sqrt{\frac{L \omega _0}{2\hbar }} Q_{\mathrm{t},c}(0) + \frac{i P_{\mathrm{t},c}(0)}{\sqrt{2\hbar L \omega _0}}. \end{aligned}$$

In actual evaluations, the expression of ${\hat{D}} (\alpha )$ given in "Methods" is useful.

Now let us consider the following transformation

24 $$\begin{aligned} \psi {^{\prime} _{\mathrm{s},n,\pm }} ({q},t) = {\hat{T}}{^\prime} ({\hat{q}},{\hat{p}},t) {\hat{D}} (\pm \alpha ) {\hat{S}} (z) \psi {^{\prime} _{n}} ({q},0) , \end{aligned}$$

where $\psi {^{\prime} _{n}} ({q},0)$ are initial wave functions in number state in the transformed system and ${\hat{T}}{^\prime}$ is a time evolution operator of the form [20, 33]

25 $$\begin{aligned} {\hat{T}}{^\prime}({\hat{q}},{\hat{p}},t) = \exp \left( - \frac{i }{\hbar }\int _0^t {\hat{H}}{^\prime}({\hat{q}}, {\hat{p}}, \tau ) d \tau \right) . \end{aligned}$$

To derive $\psi {^{\prime} _{\mathrm{s},n,\pm }} ({q},t)$ from Eq. (24), we first need the formulae $\psi {^{\prime} _{n}} ({q},0)$. By solving the Schrödinger equation with Eq. (12) in the transformed system, we easily obtain the corresponding wave functions in the number state and confirm that their initial values are given by

26 $$\begin{aligned} \psi {^{\prime} _{n}} ({q},0) = \left( \frac{L \omega _0}{\hbar \pi }\right) ^{1/4} \frac{1}{\sqrt{2^nn!}} H_n \left[ \left( \frac{L \omega _0}{\hbar }\right) ^{1/2} {q}\right] \exp \left( -\frac{L \omega _0}{2\hbar } {q}^2\right) . \end{aligned}$$

These are the same as those of the simple harmonic oscillator with the angular frequency $\omega _0$. The action of a squeezing operator in the initial number state gives

27 $$\begin{aligned}{\hat{S}} (z) \psi {^{\prime} _{n}} ({q},0) & = \left( \frac{L \omega _0}{\hbar \pi }\right) ^{1/4} \frac{1}{\sqrt{2^nn!}} \sqrt{\frac{G_{b}^n}{G_{a}}} H_n \left[ \left( \frac{L \omega _0}{\hbar G_{c}}\right) ^{1/2} {q}\right] \nonumber \\& \quad \times \exp \left( -\frac{L \omega _0}{2\hbar } G_{d} {q}^2\right) , \end{aligned}$$

where

28 $$\begin{aligned} G_{a}= & {} \cosh r + e^{i \phi } \sinh r , \end{aligned}$$

29 $$\begin{aligned} G_{b}= & {} \frac{\cosh r + e^{-i \phi } \sinh r}{ \cosh r + e^{i \phi } \sinh r} , \end{aligned}$$

30 $$\begin{aligned} G_{c}= & {} \cosh ^2 r + \hbox{sinh} ^2 r + 2\cos \phi \cosh r\sinh r , \end{aligned}$$

31 $$\begin{aligned} G_{d}= & {} \frac{1- i \sin \phi \sinh r(\cosh r + e^{i \phi } \sinh r)}{ (\cosh r + \cos \phi \sinh r)(\cosh r + e^{i \phi } \sinh r)}. \end{aligned}$$

Further action of ${\hat{D}} (\pm \alpha )$ and ${\hat{T}}{^\prime} ({\hat{q}},{\hat{p}},t)$, in turn, yields [7, 33]

32 $$\begin{aligned}\psi _{\mathrm{s},n,\pm }' ({q},t) &=\root 4 \of {\frac{L \omega _0}{\hbar \pi }} \frac{1}{\sqrt{2^nn!}} \sqrt{\frac{(h_{b} G_{b})^n}{h_{a} G_{a}}} \nonumber \\& \quad \times H_n \left [\sqrt{\frac{L \omega _0}{\hbar h_{a}^2 h_{b} G_{c}}} [{q} \mp Q_{\mathrm{t},c}(t) ]\right ] \nonumber \\&\quad \times \exp \bigg \{-\frac{L \omega _0}{2\hbar h_{a}} \bigg [ [G_{d}\cos \Omega (t) + i \sin \Omega (t) ]q^2 \nonumber \\& \qquad \qquad \mp 2q \bigg (G_{d} Q_{\mathrm{t},c}(0) + i \frac{P_{\mathrm{t},c}(0)}{\omega _0 L} \bigg ) \nonumber \\& \qquad \qquad + Q_{\mathrm{t},c}^2(0) G_{d} \cos \Omega (t) \bigg ]\bigg \} \nonumber \\&\quad \times \exp \left [ -\frac{i P_{\mathrm{t},c}^2(0) \sin \Omega (t)}{2 L \omega _0 h_{a}\hbar } - i \frac{Q_{\mathrm{t},c}(0)P_{\mathrm{t},c}(0)}{\hbar } \right. \\ & \qquad \qquad \left. \times \left ( \frac{1}{2} - i \frac{G_{d} \sin \Omega (t)}{h_{a}} \right ) \right ] \nonumber \\&\quad \times \exp \left [ -\frac{i}{\hbar } \int _0^t {\mathcal L}_{p} (\tau ) \text{ d } \tau \right ], \end{aligned}$$

where

33 $$\begin{aligned}\Omega (t) &= \rho _0^2 \omega _0 \int _0^t \frac{e^{-(R/L)\tau }}{\rho ^2 (\tau )} d\tau , \end{aligned}$$

34 $$\begin{aligned}h_{a} &= \cos \Omega (t) + i G_{d} \sin \Omega (t) , \end{aligned}$$

35 $$\begin{aligned}h_{b}& = 1- \frac{2 i \sin \Omega (t)}{h_{a} G_{c}} . \end{aligned}$$

Let us consider superposition states composed of the two DSNSs in the transformed system, which is

36 $$\begin{aligned} \psi {_{\mathrm{s},n}^{\prime \epsilon}} ({q},t) = \lambda _\mathrm{s}^\epsilon [\psi {^{\prime} _{\mathrm{s},n,+}} ({q},t) + \epsilon \psi {^{\prime} _{\mathrm{s},n,-}} ({q},t) ] , \end{aligned}$$

where $\epsilon$ is given by $\epsilon =|\epsilon |e^{i\varphi }$ and $\lambda^{\epsilon}_s$ is a normalization constant of which the formula will be derived later. The superposition states in the original system are obtained by acting ${\hat{U}}$ in these states:

37 $$\begin{aligned} \psi _{\mathrm{s},n}^\epsilon ( {q},t) = {\hat{U}} \psi {_{\mathrm{s},n} ^ {\prime \epsilon}} ( {q},t) . \end{aligned}$$

A rigorous evaluation using Eq. (11) gives

38 $$\begin{aligned} \psi _{\mathrm{s},n}^\epsilon ( {q},t) = \lambda _\mathrm{s}^\epsilon [\psi _{\mathrm{s},n,+} ( {q},t) + \epsilon \psi _{\mathrm{s},n,-} ( {q},t)], \end{aligned}$$

where

39 $$\begin{aligned}\psi _{\mathrm{s},n,\pm } ({q},t) &= \root 4 \of {\frac{L \omega _0}{\hbar \pi }} \frac{\sqrt{{\rho _0}/{\rho (t)}}}{\sqrt{2^nn!}} \sqrt{\frac{(h_{b} G_{b})^n}{h_{a} G_{a}}} H_n \left [\frac{\xi _{\pm }(q,t)}{\sqrt{h_{a}^2 h_{b} G_{c}}}\right ] \nonumber \\&\quad \times \exp \left( \frac{i}{\hbar } P_{p}(t) {q} \right) \exp \left ( \frac{i L \dot{\rho }(t)e^{(R/L)t}}{2\hbar \rho (t)} [q - Q_{p}(t)]^2 \right) \nonumber \\&\quad \times \exp \bigg \{-\frac{L \omega _0}{2\hbar h_{a}} \bigg [ [G_{d}\cos \Omega (t) + i \sin \Omega (t) ] \frac{\rho _0^2}{\rho ^2(t)}[q-Q_p(t)]^2 \nonumber \\&\qquad \mp 2\frac{\rho _0}{\rho (t)}[q-Q_p(t)] \bigg (G_{d} Q_{\mathrm{t},c}(0) + i \frac{P_{\mathrm{t},c}(0)}{\omega _0 L} \bigg ) \nonumber \\&\qquad + Q_{\mathrm{t},c}^2(0) G_{d} \cos \Omega (t) \bigg ]\bigg \} \nonumber \\ & \quad \times \exp \left [ -\frac{i P_{\mathrm{t},c}^2(0) \sin \Omega (t)}{2 L \omega _0 h_{a}\hbar } - i \frac{Q_{\mathrm{t},c}(0)P_{\mathrm{t},c}(0)}{\hbar } \times \left( \frac{1}{2} - i \frac{G_{d} \sin \Omega (t)}{h_{a}} \right) \right] \nonumber \\ &\quad \times \exp \left [ -\frac{i}{\hbar } \int _0^t {\mathcal L}_{p} (\tau ) \text{ d } \tau \right] , \end{aligned}$$

with

40 $$\begin{aligned} \xi _{\pm }(q,t) = \sqrt{\frac{L\omega _0}{\hbar }} \bigg ( \frac{\rho _0}{\rho (t)} [{q}- Q_{p}(t) ] \mp Q_{\mathrm{t},c}(t) \bigg ). \end{aligned}$$

These are the full wave functions in the superposition states of the DSNSs. We can use them to derive an expectation value of various quantum observables in the superposition states.

From the absolute square of Eq. (38), we also have the probability densities as

41 $$\begin{aligned} \left| \psi _{\mathrm{s},n}^{\epsilon }( {q},t)\right| ^2 &= \sqrt{\frac{L\omega _0}{\hbar \pi }} \frac{1}{2^n n!}\frac{ \rho _0}{\rho (t)d } |\lambda _\mathrm{s}^\epsilon |^2 \nonumber \\ &\quad \times \exp \left \{ - \frac{L \omega _0 }{\hbar d^2 } \left [\frac{\rho _0^2}{\rho ^2(t)}[q-Q_p(t)]^2 +Q_{\mathrm{t},c}^2 (t) \right ] \right \} \nonumber \\&\quad \times \left \{ e^{Z(q,t)} \left[H_n \bigg (\frac{\xi _{+}(q,t)}{d}\bigg )\right]^2 + |\epsilon |^2 e^{-Z(q,t)}\left [H_n \bigg (\frac{\xi _{-}(q,t)}{d}\bigg )\right ]^2 \right. \nonumber \\&\qquad \left. +\, 2 |\epsilon |H_n \bigg (\frac{\xi _{+}(q,t)}{d}\bigg )H_n \bigg (\frac{\xi _{-}(q,t)}{d}\bigg ) \right. \nonumber \\ & \left. \qquad \times\, {\text{cos}} \left [\frac{2B(t)}{\hbar d^2 } \frac{\rho _0}{\rho (t)}[q-Q_p(t)] -\varphi \right ] \right \} , \end{aligned}$$

where

42 $$\begin{aligned}d^2 &= \cosh (2r) +\cos [2\Omega (t)-\phi ] \sinh (2r) \nonumber \\& = {s_0^2 \cos ^2 [\Omega ( t)- \phi /2] + s_0^{-2} \sin ^2 [\Omega ( t)}-\phi /2] , \end{aligned}$$

43 $$\begin{aligned}s_0 &= \cosh r + \sinh r = e^{r}, \end{aligned}$$

44 $$\begin{aligned}&Z(q,t) = \frac{2L\omega _0 Q_{\mathrm{t},c}(t) }{\hbar d^2}\frac{\rho _0}{\rho (t)}[q-Q_p(t)], \end{aligned}$$

45 $$\begin{aligned}B(t) &= P_{\mathrm{t},c}(t)\cosh (2r)+ \{ P_{\mathrm{t},c}(0) \cos [\Omega (t)-\phi ] \nonumber \\&\quad + L\omega _0 Q_{\mathrm{t},c}(0) \sin [\Omega (t) -\phi ] \} \sinh (2r). \end{aligned}$$

It is possible to represent *d* in terms of *s* given in Eq. (21) instead of $s_0$. The transformation relation from $s_0$ to *s* and vice versa are

46 $$\begin{aligned} s_0\rightarrow & {} \frac{s+\sqrt{s^2-\sin ^2 \phi }}{1+\cos \phi } , \end{aligned}$$

47 $$\begin{aligned} s\rightarrow & {} \frac{1-\cos \phi }{2s_0} + \frac{s_0(1+\cos \phi )}{2} . \end{aligned}$$

Now, the formula of $\lambda _\mathrm{s}^\epsilon$ is derived from the normalization condition, $\int _{-\infty }^\infty |\psi _{\mathrm{s},n}^{\epsilon }( {q},t)|^2 dq =1$. It results in

48 $$\begin{aligned} |\lambda _\mathrm{s}^\epsilon |^2 = \{1+ |\epsilon |^2 +2 |\epsilon | \exp (-\eta (t) ) L_n [2\eta (t)] \cos \varphi \}^{-1}, \end{aligned}$$

where

49 $$\begin{aligned} \eta (t) = \frac{1}{\hbar d^2} \left( L\omega _0 Q_{\mathrm{t},c}^2 (t) + \frac{B^2(t)}{L\omega _0} \right) . \end{aligned}$$

If we put $r=0$, Eq. (48) reduces to Eq. (26) of Ref. [20], which is related to the superposition of DNSs. The probability densities, Eq. (41), consist of three terms. The first two terms correspond to the densities associated to $\psi _{\mathrm{s},n,+} ({q},t)$ and $\psi _{\mathrm{s},n,-} ({q},t)$, respectively. The last term that is represented in terms of a cosine function exhibits interference between the two components. This term signifies nonclassical properties of the quantum system, that do not appear in the counterpart classical system. The interference term is especially large when the two wave packets associated with $\psi _{\mathrm{s},n,+} ({q},t)$ and $\psi _{\mathrm{s},n,-} ({q},t)$ meet in space. From Eq. (41), we can confirm that the width of the packet is determined by the value of *d*. If $d< 1$, the packet corresponds to that of the *q*-squeezing case, whereas if $d> 1$, the packet belongs to that of the *p*-squeezing case. However, the degree of squeezing varies more or less with time according to the time variation of *d*.

For the case that *z* is real ($\phi = 0$), Eqs. (42) and (49) reduce to

50 $$\begin{aligned}&d_0^2 = {s_0^2 \cos ^2 \Omega ( t) + s_0^{-2} \sin ^2 \Omega ( t)}, \end{aligned}$$

51 $$\begin{aligned}&\eta _0 = \frac{1}{\hbar } \left( s_0^{-2}L\omega _0 Q_{\mathrm{t},c}^2 (0) + s_0^2 \frac{P_{\mathrm{t},c}^2(0)}{L\omega _0} \right) . \end{aligned}$$

We see that $d_0$ varies with time for an arbitrary value of $s_0$, while $\eta _0$ is constant. However, for the case that $s_0=1$, $d_0$ does not vary with time and reduces to unity that corresponds to the situation of no squeezing at all.

Note that $\eta _0$ can be rewritten in a simple form as

52 $$\begin{aligned} \eta _0 = 2 |f|^2 , \end{aligned}$$

where

53 $$\begin{aligned} f = \alpha \cosh r - \alpha ^* \sinh r . \end{aligned}$$

In fact, Eq. (53) is slightly different from that of the system proposed by El-Orany et al. (e.g. see Eq. (4) of Ref. [31]).

For the case that $n=0$, $\epsilon = \pm 1$, and $\phi = 0$, the probability density becomes

54 $$\begin{aligned}\left| \psi _{\mathrm{s},n=0}^{\epsilon = \pm 1}( {q},t)\right| ^2 &= \sqrt{\frac{L\omega _0}{\hbar \pi }} \frac{2 \rho _0}{\rho (t)d_0 } \left| \lambda _{0,\mathrm s}^{\epsilon =\pm 1}\right| ^2 \exp \left \{ - \frac{L \omega _0 }{\hbar d_0^2 }\left [\frac{\rho _0^2}{\rho ^2(t)} [q-Q_p(t)]^2 +Q_{\mathrm{t},c}^2 (t) \right ] \right \} \nonumber \\&\quad \times \left \{ \cosh \left [ \frac{2L\omega _0 Q_{\mathrm{t},c}(t) }{\hbar d_0^2} \frac{\rho _0}{\rho (t)}[q-Q_p(t)] \right ] \pm \cos \left [ \frac{2}{\hbar d_0^2 s_0^2}\frac{\rho _0}{\rho (t)}[q-Q_p(t)] \right. \right. \nonumber \\&\quad \left. \Bigg. \times [P_{\mathrm{t},c}(0)s_0^4 \cos \Omega ( t) - L\omega _0 Q_{\mathrm{t},c} (0) \sin \Omega ( t)] \Bigg ] \right\} , \end{aligned}$$

where $\lambda _{0, \mathrm s}^{\epsilon } = \lambda _\mathrm{s}^{\epsilon } |_{\phi =0}$. We can see from this expression that, for the non-squeezing case ($s_0=1$), the time dependence of phase of the interference term follows $[q-Q_p(t)]P_{\mathrm{t},c}(t)/\rho (t)$.

For a further simplified case which falls under $n=0$, $\epsilon = \pm 1$, $\mathcal {E}(t) =0$, $R=0$, and $C(t) = C(0)$, we confirm that $\Omega (t) \rightarrow \omega _0 t$ and the probability density reduces to

55 $$\begin{aligned}\left| \psi _{\mathrm{s},n=0}^{\epsilon = \pm 1}( {q},t)\right| ^2 &= \sqrt{\frac{L\omega _0}{\hbar \pi }} \frac{2}{d_0} \left| {\lambda _{0,\mathrm s}^{\epsilon =\pm 1}}\right| ^2 \exp \bigg ( - \frac{L \omega _0 [q^2 +Q_{\mathrm{t},c}^2 (t) ]}{\hbar d_0^2 } \bigg ) \nonumber \\&\quad \times \bigg \{ \cosh \bigg [ \frac{2L\omega _0 Q_{\mathrm{t},c}(t) q}{\hbar d_0^2} \bigg ] \pm \cos \bigg [ \frac{2q}{\hbar d_0^2 s_0^2}[P_{\mathrm{t},c}(0)s_0^4 \cos (\omega _0 t) \nonumber \\&\quad - L\omega _0 Q_{\mathrm{t},c}(0) \sin (\omega _0 t)] \bigg ] \bigg \}. \end{aligned}$$

In this case, the phase of the interference term is determined by $q[P_{\mathrm{t},c}(0)s_0^2\cos (\omega _0 t) - L\omega _0 Q_{\mathrm{t},c}(0) \sin (\omega _0 t)/s_0^2]$. For the non-squeezing case, it is fixed simply by $q P_{\mathrm{t},c}(t)$.

**Fig. 1:**
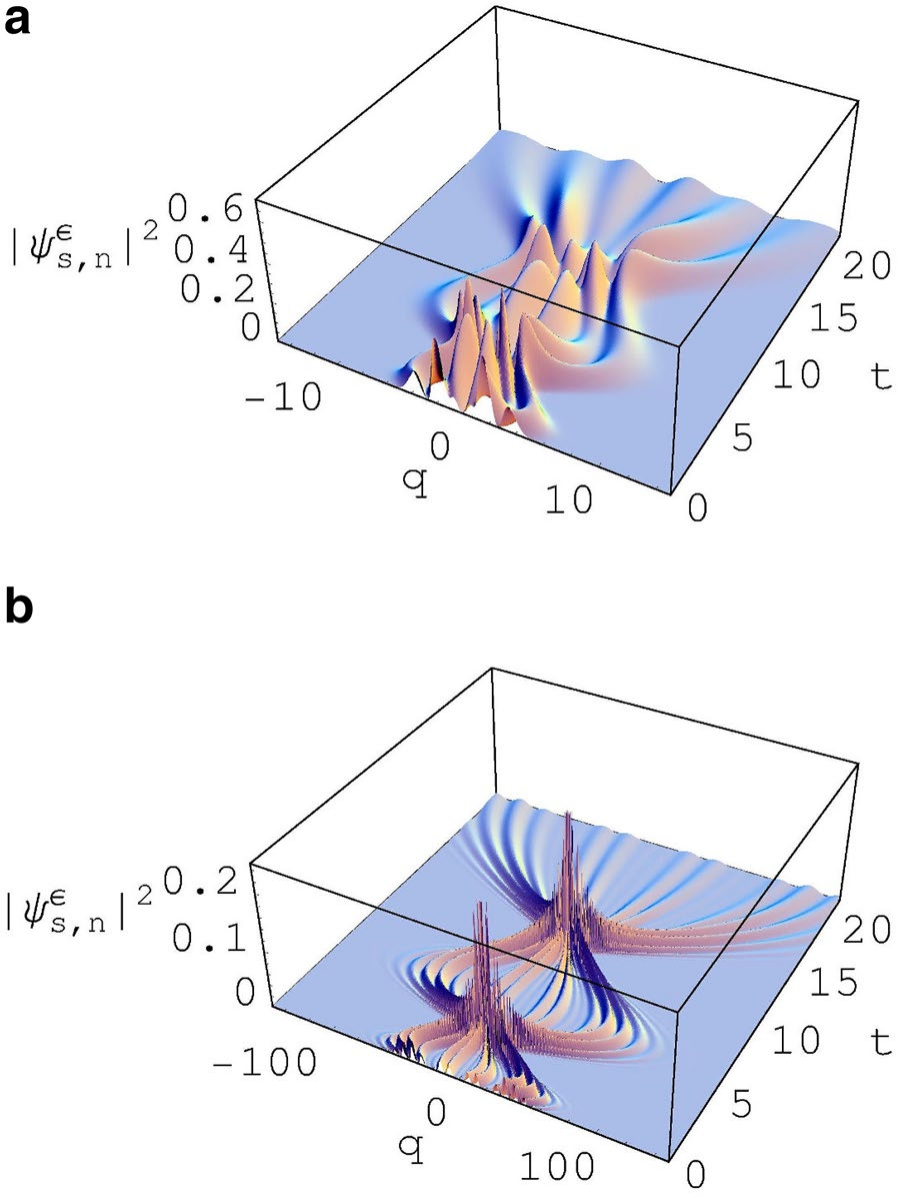
The probability density $|\psi _{\mathrm{s},n}^\epsilon ({q},t)|^2$ [Eq. (41)] plotted under the choice of parameters as illustrated in Eqs. (81)–(87) as a function of *q* and *t*. Here, we have chosen relatively small values of $Q_{\mathrm{t},c}(0)$ and $P_{\mathrm{t},c}(0)$ and they are ($Q_{\mathrm{t},c}(0)$, $P_{\mathrm{t},c}(0)$) = (1, 1), while we have assumed that the electromotive force is zero, $({\textsf {Q}}, \omega _1)=(0, 0)$. The squeezing parameter *r* is 0.3 for (**a**) and 3 for (**b**). Other values taken here are $L=1$, $C_0 = 1$, $\omega _0=1$, $\epsilon =(1+i)/\sqrt{2}$, $\phi =1$, $\hbar =1$, $\beta =0.1$, and $n=3$

**Fig. 2:**
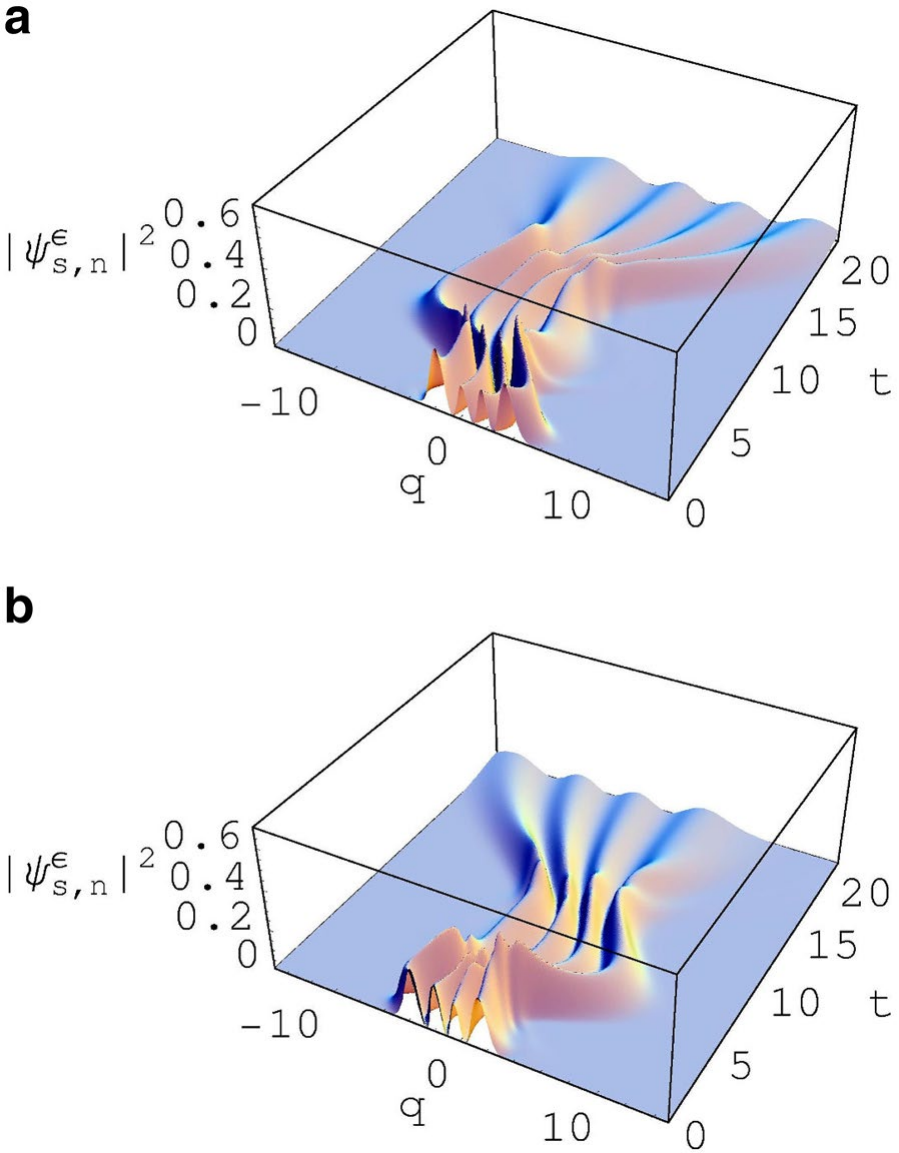
Asymmetric evolution of the probability density $|\psi _{\mathrm{s},n}^\epsilon ({q},t)|^2$ given in Eq. (41). The relative amplitude $\epsilon$ associated to $\psi _{\mathrm{s},n,-} ({q},t)$ is $0.1\epsilon _0$ for (**a**) and $10\epsilon _0$ for (**b**) where $\epsilon _0=(1+i)/\sqrt{2}$. The conditions given in Eqs. (81)–(87) are used. The values of other parameters taken here are $L=1$, $C_0 = 1$, $\omega _0=1$, $Q_{\mathrm{t},c}(0)=1$, $P_{\mathrm{t},c}(0)=1$, $r=0.3$, $\phi =1$, $\hbar =1$, $\beta =0.1$, $n=3$, and ${\textsf {Q}}=0$, which, in fact, are the same as those of Fig. 1a

**Fig. 3:**
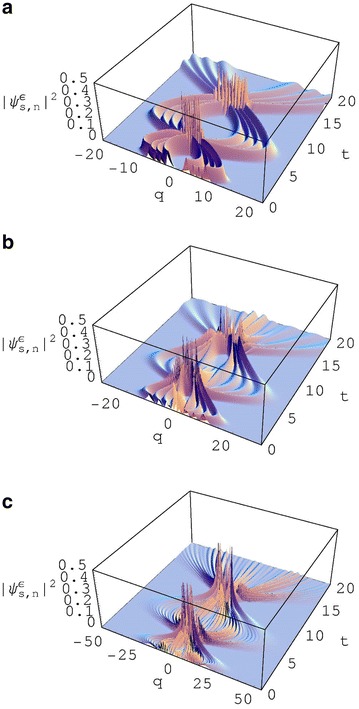
The probability density $|\psi _{\mathrm{s},n}^\epsilon ({q},t)|^2$ [Eq. (41)] plotted under the choice of parameters as illustrated in Eqs. (81)–(87) as a function of *q* and *t*. Here, we have chosen relatively large values of $Q_{\mathrm{t},c}(0)$ and $P_{\mathrm{t},c}(0)$ and they are ($Q_{\mathrm{t},c}(0)$, $P_{\mathrm{t},c}(0)$) = (5, 5), while we have assumed that the electromotive force is zero, $({\textsf {Q}}, \omega _1)=(0, 0)$. The squeezing parameter *r* is 0.3 for (**a**), 1 for (**b**), and 2 for (**c**). Other values taken here are $L=1$, $C_0 = 1$, $\omega _0=1$, $\epsilon =(1+i)/\sqrt{2}$, $\phi =1$, $\hbar =1$, $\beta =0.1$, and $n=3$, which, in fact, are the same as those of Fig. 1

**Fig. 4:**
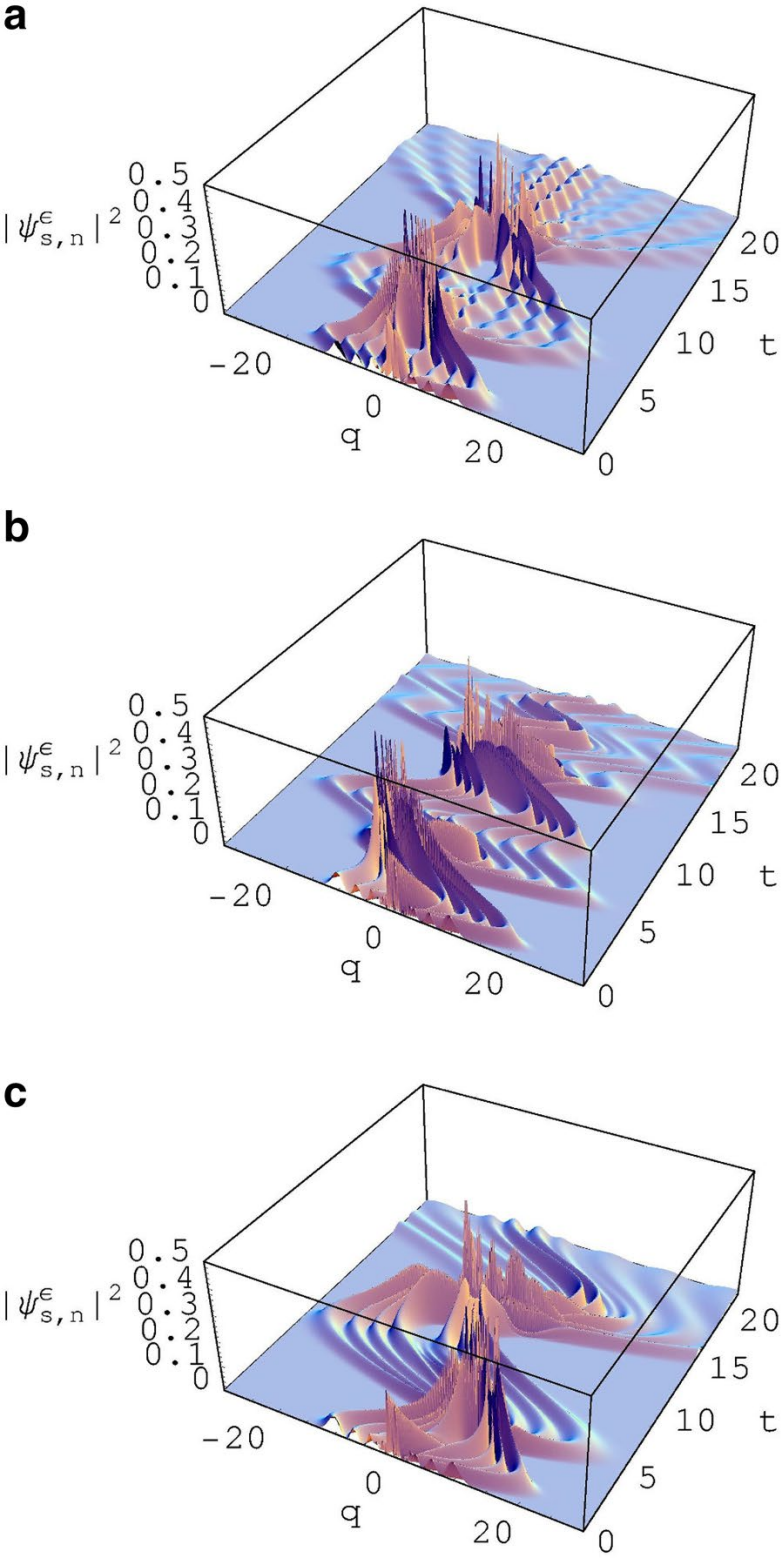
The effects of the electromotive force on the probability density $|\psi _{\mathrm{s},n}^\epsilon ({q},t)|^2$ [Eq. (41)]. We used the parameters illustrated in Eqs. (81)–(87). The values of parameters $({\textsf {Q}}, \omega _1)$ associated with the electromotive force are $({\textsf {Q}}, \omega _1)=(0.5, 5)$ for (**a**), $({\textsf {Q}}, \omega _1)=(5, 1.5)$ for (**b**), and $({\textsf {Q}}, \omega _1)=(10, 0.5)$ for (**c**). Other values taken here are $Q_{\mathrm{t},c}(0)=5$, $P_{\mathrm{t},c}(0)=5$, $L=1$, $C_0 = 1$, $\omega _0=1$, $\epsilon =(1+i)/\sqrt{2}$, $r=1$, $\phi =1$, $\hbar =1$, $\beta =0.1$, and $n=3$, which, in fact, are the same as those of Fig. 3b

The probability density $|\psi _{\mathrm{s},n}^\epsilon ( {q},t)|^2$ given in Eq. (41) is plotted in Figs. 1, 2, 3, and 4 as a function of *q* and *t* under the choice of parameters as Eqs. (81)–(87) in "Methods". The probability density given in Fig. 1 is for relatively small displacing parameters: $(Q_{\mathrm{t},c}(0), P_{\mathrm{t},c}(0)) =(1,1)$. By comparing Fig. 1a and b with each other, we can confirm that the width of the wave function becomes large as the value of *r* increases. Figure 1a is *q*-squeezing and Fig. 1b is *p*-squeezing. Figure 2a shows the time evolution of the wave packet that is dominated by $\psi _{\mathrm{s},n,+} ( {q},t)$, while Fig. 2b is the case that $\psi _{\mathrm{s},n,-} ( {q},t)$ is dominant. By adding these two packets, we may roughly obtain the normal wave packet, which is, for example, the one given in Fig. 1, with equal contribution of the two components. Figure 3 is the probability density for sufficiently high displacing parameters: $(Q_{\mathrm{t},c}(0), P_{\mathrm{t},c}(0)) =(5,5)$. We see by comparing Fig. 3a with Fig. 1a that the displacement of the wave packet is quite distinct when the displacing parameters are large, as expected. The effects of a driving electromotive force on the circuit can be identified from Fig. 4. The wave packets in Fig. 4 are distorted more or less significantly, due to the influence of a time-dependent electromotive force.

We can find nonclassical features of the system from quantum interference displayed in Figs. 1, 2, 3, and 4. Highly peaked ripples in density, formed near the center ($q=0$), signifies nonclassicality of the superposition state. The distribution of *q* exhibits peaks at the spots where the two or more non-zero line-shaped distributions intersect with each other. The distribution-lines are oscillating and mainly composed of two groups according to the two individual components of the superposition state. In the same situation, the distribution of the conjugate variable *p* oscillates as a reflection of quantum interference [34].

Notice that the concepts of the superposition principle and quantum interference are applied to developing fundamental mechanisms for quantum information theory. In connection with this, quantum physics and quantum field theory are undergoing a time of revolutionary change in these days.

### Time evolution of quantum observables

The time evolution of quantum observables in the quantum states developed in the previous sections can be found by evaluating the expectation value of them. Let us see for example the time evolution of charges and currents in the DSNS in the original system. Considering the fact that the notation of the wave functions in this state can be rewritten, without loss of generality, in the form

56 $$\begin{aligned} \psi _{\mathrm{s},n}^\epsilon (q,t) = \langle q | \psi _{\mathrm{s},n}^\epsilon (t) \rangle , \end{aligned}$$

and using the consecutive unitary transformation, the expectation value of an arbitrary quantum observable $\hat{\mathcal O}$ can be evaluated from

57 $$\begin{aligned} \langle \psi _{\mathrm{s},n}^\epsilon (t) | \hat{\mathcal O} | \psi _{\mathrm{s},n}^\epsilon (t) \rangle = |\lambda _\mathrm{s}^\epsilon |^2 \langle \psi _{n}'(0) | \hat{\mathcal O}' | \psi {^{\prime} _{n}} (0) \rangle , \end{aligned}$$

where

58 $$\begin{aligned} \hat{\mathcal O}' = {\hat{S}}^\dagger [{\hat{D}}^\dagger (\alpha )+\epsilon ^* {\hat{D}}^\dagger (-\alpha )] {\hat{T}}{^{\prime \dagger}} {\hat{U}}^\dagger \hat{\mathcal O} {\hat{U}} {\hat{T}}{^\prime} [{\hat{D}}(\alpha )+\epsilon {\hat{D}}(-\alpha )] {\hat{S}} . \end{aligned}$$

Let us perform successive operations given in Eq. (58) after replacing $\hat{\mathcal O}$ with ${\hat{q}}$ and ${\hat{p}}$. Then, with the use of Eqs. (67)–(80) in "Methods", we have

59 $$\begin{aligned}{\hat{q}}{^\prime} &= \sqrt{\frac{\hbar }{2L\omega _0}} \frac{\rho (t)}{\rho _0} \{({\hat{b}}+\alpha ) e^{-i\Omega (t)} +({\hat{b}}^\dagger +\alpha ^*) e^{i\Omega (t)} \nonumber \\&\quad +\epsilon [({\hat{b}}+\alpha )e^{2(\alpha ^* {\hat{b}}-\alpha {\hat{b}}^\dagger )}e^{-i\Omega (t)} +({\hat{b}}^\dagger +\alpha ^*)e^{2(\alpha ^* {\hat{b}}-\alpha {\hat{b}}^\dagger )}e^{i\Omega (t)} ] \nonumber \\&\quad +\epsilon ^*[({\hat{b}}-\alpha )e^{-2(\alpha ^* {\hat{b}}-\alpha {\hat{b}}^\dagger )}e^{-i\Omega (t)} +({\hat{b}}^\dagger -\alpha ^*)e^{-2(\alpha ^* {\hat{b}}-\alpha {\hat{b}}^\dagger )}e^{i\Omega (t)} ] \nonumber \\&\quad +|\epsilon |^2 [({\hat{b}}-\alpha ) e^{-i\Omega (t)} +({\hat{b}}^\dagger -\alpha ^*) e^{i\Omega (t)}] \} + Q_p(t), \end{aligned}$$

60 $$\begin{aligned}{\hat{p}}{^\prime} &= F(t) ({\hat{b}}+\alpha ) e^{-i\Omega (t)} +F^*(t)({\hat{b}}^\dagger +\alpha ^*) e^{i\Omega (t)} \nonumber \\&\quad +\epsilon [F(t)({\hat{b}}+\alpha )e^{2(\alpha ^* {\hat{b}}-\alpha {\hat{b}}^\dagger )}e^{-i\Omega (t)} +F^*(t)({\hat{b}}^\dagger +\alpha ^*)e^{2(\alpha ^* {\hat{b}}-\alpha {\hat{b}}^\dagger )}e^{i\Omega (t)} ] \nonumber \\&\quad+\epsilon ^*[F(t)({\hat{b}}-\alpha )e^{-2(\alpha ^* {\hat{b}}-\alpha {\hat{b}}^\dagger )}e^{-i\Omega (t)} +F^*(t)({\hat{b}}^\dagger -\alpha ^*)e^{-2(\alpha ^* {\hat{b}}-\alpha {\hat{b}}^\dagger )}e^{i\Omega (t)} ] \nonumber \\&\quad +|\epsilon |^2 [F(t)({\hat{b}}-\alpha ) e^{-i\Omega (t)} +F^*(t)({\hat{b}}^\dagger -\alpha ^*) e^{i\Omega (t)}] + P_p(t), \end{aligned}$$

where ${\hat{b}}$ and ${\hat{b}}$ ^†^ are defined in "Methods" [see Eqs. (79) and (80)] and

61 $$\begin{aligned} F(t) = \sqrt{\frac{\hbar L}{2}} \left( \frac{\dot{\rho }(t)}{\rho _0 \sqrt{\omega _0}}e^{(R/L)t} - i \frac{\rho _0}{\rho (t)}\sqrt{\omega _0} \right) . \end{aligned}$$

Let us calculate the expectation value of canonical variables with an assumption that $\alpha$ is sufficiently small than unity. If we consider up to $[\alpha ^{(*)}]^3$ terms, the expectation values are given by

62 $$\begin{aligned}\langle \psi _{\mathrm{s},n}^\epsilon (t) | {\hat{q}} | \psi _{\mathrm{s},n}^\epsilon (t) \rangle &=|\lambda _\mathrm{s}^\epsilon |^2\sqrt{\frac{\hbar }{2L\omega _0}} \frac{\rho (t)}{\rho _0} \{ (1-\epsilon ^*\epsilon ) [\alpha e^{-i\Omega (t)}+\alpha ^* e^{i\Omega (t)} ] \nonumber \\&\quad + (\epsilon -\epsilon ^*) [ { K} e^{-i\Omega (t)} - { K}^* e^{i\Omega (t)} ] \} +Q_p(t), \end{aligned}$$

63 $$\begin{aligned}\langle \psi _{\mathrm{s},n}^\epsilon (t) | {\hat{p}} | \psi _{\mathrm{s},n}^\epsilon (t) \rangle &=|\lambda _\mathrm{s}^\epsilon |^2 \{(1-\epsilon ^*\epsilon ) [F(t)\alpha e^{-i\Omega (t)}+F^*(t)\alpha ^* e^{i\Omega (t)} ] \nonumber \\&\quad + (\epsilon -\epsilon ^*) [F(t) { K} e^{-i\Omega (t)} -F^*(t) { K}^* e^{i\Omega (t)} ]\} +P_p(t), \end{aligned}$$

where

64 $$\begin{aligned}{ K} &= 2\{\alpha ^* e^{i\phi } (2n+1)\cosh r \sinh r -\alpha [(n+1)(\cosh r)^2+n(\sinh r)^2- 1/2]\} \nonumber \\&\quad +4\alpha ^{*3} e^{2i\phi } (2n^2 + 2n +1)(\cosh r)^2(\sinh r)^2 +2\alpha ^{*2}\alpha e^{i\phi } \{ (2n+1)\cosh r \sinh r \nonumber \\&\quad -2 [(3n^2+4n+2)(\cosh r)^3 \sinh r + (3n^2+2n+1)\cosh r(\sinh r)^3] \} \nonumber \\&\quad +2\alpha ^* \alpha ^2 \{ -(2n+1)[(\cosh r)^2+(\sinh r)^2] +2[(n^2+2n+1)(\cosh r)^4 \nonumber \\&\quad +n^2(\sinh r)^4 +(4n^2+4n+2)(\cosh r)^2(\sinh r)^2 ] \} +2\alpha ^3 e^{-i\phi } \{ (2n+1) \nonumber \\&\quad \times \cosh r \sinh r-2 [(n^2+2n+1)(\cosh r)^3 \sinh r + n^2\cosh r(\sinh r)^3 ] \} . \end{aligned}$$

**Fig. 5:**
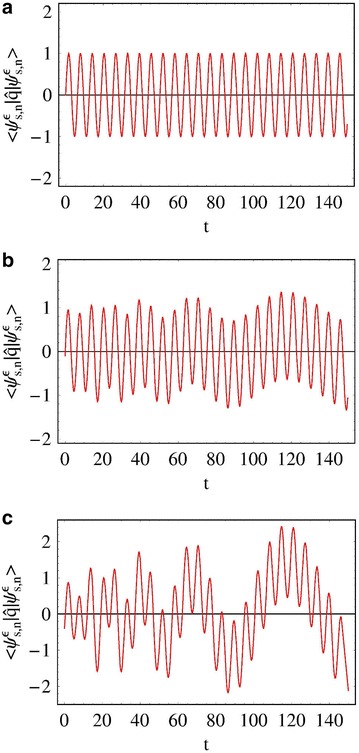
Time evolution of the expectation value $\langle \psi _{\mathrm{s},n}^\epsilon (t) | {\hat{q}} | \psi _{\mathrm{s},n}^\epsilon (t) \rangle$ for the case that time functions are chosen to be Eqs. (81)–(87). The value of $\epsilon$ is 1 for (**a**), $1+0.005i$ for (**b**), and $1+0.02i$ for (**c**). Other values taken here are $L=1$, $C_0 = 1$, ${\textsf {Q}}=1$, $\omega _{1}=1$, $\rho _0=1$, $r =1$, $\phi =1$, $Q_{\mathrm{t},c}(0)=1$, $P_{\mathrm{t},c}(0)=1$, $\hbar =1$, $\beta =0.02$, and $n=1$

To see the time behavior of canonical variables, let us see again for the case given in Eqs. (81)–(87). From Fig. 5, we can confirm that the expectation value of ${\hat{q}}$ is more or less distorted with time due to the influence of the time dependence of parameters.

## Conclusions

Squeezing effects of a series RLC nanoelectronic circuit of which parameters depend on time are investigated focusing on the nonclassical properties of its superposition states. Due to the time dependence of the Hamiltonian, the system is classified as a kind of time-dependent Hamiltonian system (TDHS) that requires an alternative rigorous treatment for the study of their quantum properties. Hence, we introduced a quadratic invariant operator which is very useful when we study quantum features of a TDHS. Because the Hamiltonian (or the invariant operator) of the system is somewhat complicated, we had transformed the Hamiltonian to a simple form by using a unitary operator. We confirmed that the wave functions in the transformed system are the same as those of the simple harmonic oscillator. We considered the superposition states composed of two distinct DSNSs in the transformed system. By inverse transformation of quantum solutions (wave functions) identified in the transformed system, we also obtained the full wave functions for the corresponding superposition states in the original system [see Eq. (38) with Eqs. (39) and (40)]. On the basis of these solutions, we investigated quantum characteristics of the superposition states.

The time evolutions of probability densities in superposition states are illustrated using corresponding graphics. It is confirmed by comparing Figs. 3a with 1a that the effects of displacement are conspicuous when the initial values $Q_{\mathrm{t},c}(0)$ and $P_{\mathrm{t},c}(0)$ are large. For the case that *C*(*t*) is given by Eq. (81), the effects of displacing become less prominent as the value of $\beta$ increases. When the driving electromotive power source is exerted on the system, the wave packet of charges exhibits a significant distortion. We have also confirmed that the time evolution of the expectation value of ${\hat{q}}$ is more or less distorted on account of the effects of time dependence of parameters. The width of the packet for the superposition of the DSNSs becomes large as the squeezing parameter *r* increases. By analyzing the exponential function given in Eq. (41), we conclude that the squeezing effect depends on *d*. The packet exhibits the *q*-squeezing for $d< 1$ whereas the *p*-squeezing for $d> 1$. If we consider the fact that *d* varies more or less over time [see Eq. (42)], the degree of squeezing is not constant.

Some interference structures in the superposition states have appeared when the two distinct component states meet together in space. We can see the interference structures from Figs. 1, 2, 3, and 4, which took place near the spot of $q=0$. These indicate novel characteristics of the superposition states that can not be explained on the basis of classical mechanics. Such interference is a kind of nonclassical property like antibunching, sub-Poissonian statistics, and various others as well. In particular, these interferences bring about negativity of the Wigner function in phase space, which is a strong signature of nonclassicality for the state. Recent reports [35, 36] indicate that the nonclassical effects of dynamical systems play an important role on upcoming quantum information science performing computational and cryptographic tasks on the basis of a fantastic paradigm essentially different from traditional ones. Thanks to the technical and theoretical advancement relevant to this, new ways for manipulating difficult computational tasks will be opened, leading to a dramatical change of our way to view information.

Extensive research for the nonclassicality of superposition states have been carried out over the years and several schemes for producing such states have been proposed. The experimental observation of quantum interferences is a difficult task because the superposition of the two distinct states is apt to reduce to a simple mixture during measurements. One of the possible schemes in this line is to observe quantum interference by amplifying the states in a phase-sensitive manner [34]. Apparently, quantum mechanics is a pivotal achievement of modern physics where the novel outcomes of quantization, such as the superposition principle and interference are verified by the elegant experimental observations associated with nonclassical states of quantum systems. Theoretical developments for manipulating quantum phenomena is crucial for the proper analysis of nanoscale systems like nanoelectronic circuits which we have treated here.

## Methods

Some of mathematical formulae that are useful for deriving several results in the text are provided here.

*Mathematical Formulae A*: The following identity is necessary for deriving Eq. (20) [20, 37]

65 $$\begin{aligned}\exp \left( {\frac{1}{2\hbar } [a{\hat{q}}^2 + ic (\hat{q}\hat{p} + \hat{p}\hat{q}) - b{\hat{p}}^2]} \right) & =\frac{1}{[\cosh \theta - ({c}/{\theta }) \sinh \theta ]^{1/2}}\nonumber \\& \quad \times \exp \left({ \frac{a}{2\theta \hbar }\frac{\sinh \theta }{\cosh \theta - ({c}/{\theta }) \sinh \theta}{\hat{q}}^2} \right) \nonumber \\& \quad \times \exp \left(-\frac{i}{\hbar } \ln \left[{ \cosh \theta - ({c}/{\theta }) \sinh\theta} \right] {\hat{q}}{\hat{p}} \right) \nonumber \\& \quad\times \exp \left( {- \frac{b}{2\theta \hbar } \frac{\sinh \theta}{\cosh \theta - ({c}/{\theta }) \sinh \theta }{\hat{p}}^2}\right) , \end{aligned}$$

where $$\theta =( c^2 - ab)^{1/2}$$.

*Mathematical Formulae B*: An alternative representation of the displacement operator is given by [38]

66 $$\begin{aligned} {\hat{D}} (\alpha ) = \exp \left( -i\frac{Q_{\mathrm{t},c}(0)P_{\mathrm{t},c}(0)}{2\hbar }\right) \exp \left( i\frac{P_{\mathrm{t},c}(0) {\hat{q}}}{\hbar }\right) \exp \left( -i\frac{{Q}_{\mathrm{t},c} (0) {\hat{p}}}{\hbar }\right) . \end{aligned}$$

Sometimes, when unfolding the quantum theory, this representation is more useful than the one given in Eq. (22).

*Mathematical Formulae C*: To derive Eqs. (59) and (60) from Eq. (58), we need several successive unitary transformations. At first, the unitary transformations of the canonical variables by ${\hat{U}}$ are

67 $$\begin{aligned} {\hat{U}}^\dagger {\hat{q}} {\hat{U}}= & {} \frac{\rho (t)}{\rho _0}{\hat{q}} + Q_p(t) , \end{aligned}$$

68 $$\begin{aligned} {\hat{U}}^\dagger {\hat{p}} {\hat{U}}= & {} \frac{\rho _0}{\rho (t)}{\hat{p}} + \frac{L \dot{\rho }(t)}{\rho _0}e^{(R/L)t}{\hat{q}} + P_p(t). \end{aligned}$$

For evaluating the time evolution of the canonical variables, we can use the following formulas

69 $$\begin{aligned} {\hat{T}}^{\prime \dagger} {\hat{q}} {\hat{T}}^{\prime} = \sqrt{\frac{\hbar}{2L \omega _0}} [{\hat{a}}^{\dagger} (0)e^{i \Omega (t)} + {\hat{a}}(0)e^{-i\Omega (t)}] , \end{aligned}$$

70 $$\begin{aligned} {\hat{T}}^{\prime \dagger} {\hat{p}} {\hat{T}}^{\prime} = i\sqrt{\frac{\hbar L \omega _0}{2}} [{\hat{a}}^{\dagger} (0)e^{i\Omega (t)} -{\hat{a}}(0)e^{-i\Omega (t)}]. \end{aligned}$$

Further, we need the algebra of the forms [39]

71 $$\begin{aligned}&{\hat{D}}^\dagger (\alpha ) {\hat{a}} {\hat{D}} (\alpha ) = {\hat{a}} + \alpha , \end{aligned}$$

72 $$\begin{aligned}&{\hat{D}}^\dagger (\alpha ) {\hat{a}} {\hat{D}} (-\alpha ) = ({\hat{a}} + \alpha ) e^{2(\alpha ^* {\hat{a}}-\alpha {\hat{a}}^\dagger )}, \end{aligned}$$

73 $$\begin{aligned}&{\hat{D}}^\dagger (-\alpha ) {\hat{a}} {\hat{D}} (\alpha ) = ({\hat{a}} - \alpha ) e^{-2(\alpha ^* {\hat{a}}-\alpha {\hat{a}}^\dagger )}, \end{aligned}$$

74 $$\begin{aligned}&{\hat{D}}^\dagger (-\alpha ) {\hat{a}} {\hat{D}} (-\alpha ) = {\hat{a}} - \alpha , \end{aligned}$$

75 $$\begin{aligned}&{\hat{D}}^\dagger (\alpha ) {\hat{a}}^\dagger {\hat{D}} (\alpha ) = {\hat{a}}^\dagger + \alpha ^* , \end{aligned}$$

76 $$\begin{aligned}&{\hat{D}}^\dagger (\alpha ) {\hat{a}}^\dagger {\hat{D}} (-\alpha ) = ({\hat{a}}^\dagger + \alpha ^*) e^{2(\alpha ^* {\hat{a}}-\alpha {\hat{a}}^\dagger )}, \end{aligned}$$

77 $$\begin{aligned}&{\hat{D}}^\dagger (-\alpha ) {\hat{a}}^\dagger {\hat{D}} (\alpha ) = ({\hat{a}}^\dagger - \alpha ^*) e^{-2(\alpha ^* {\hat{a}}-\alpha {\hat{a}}^\dagger )}, \end{aligned}$$

78 $$\begin{aligned}&{\hat{D}}^\dagger (-\alpha ) {\hat{a}}^\dagger {\hat{D}} (-\alpha ) = {\hat{a}}^\dagger - \alpha ^* . \end{aligned}$$

In addition, the final step requires

79 $$\begin{aligned}&{\hat{S}}^\dagger (z) {\hat{a}} {\hat{S}} (z) = {\hat{a}}{\hbox{cosh }} r + e^{i\phi } {\hat{a}}^\dagger {\hbox{sinh }} r \equiv {\hat{b}} , \end{aligned}$$

80 $$\begin{aligned}&{\hat{S}}^\dagger (z) {\hat{a}}^\dagger {\hat{S}} (z) = {\hat{a}}^\dagger {\hbox{cosh }}r + e^{-i\phi } {\hat{a}} {\hbox{sinh }}r \equiv {\hat{b}}^\dagger . \end{aligned}$$

*Time-Dependent Parameters*: For the understanding of our development, let us consider a solvable case that is characterized by the parameters of the form [20]

81 $$\begin{aligned} C(t)= C_0 (1+\beta t)^4 , \end{aligned}$$

82 $$\begin{aligned} \mathcal {E}(t)={\textsf {Q}}\left( \frac{1}{C_0(1+\beta t)^4}-\omega _1^2 L\right) \sin (\omega _1 t ), \end{aligned}$$

83 $$R= 0 ,$$

where $C_0=C(0)$, $\beta$, ${\textsf {Q}}$, and $\omega _1$ are real constants. Under these choices, we have

84 $$\begin{aligned} \rho (t)= \rho _0 (1+\beta t), \end{aligned}$$

85 $$\begin{aligned} \Omega (t)= \frac{1}{\sqrt{LC_0}} \frac{t}{1+\beta t}, \end{aligned}$$

86 $$\begin{aligned} Q_p(t)= {\textsf {Q}} \sin (\omega _1 t), \end{aligned}$$

87 $$\begin{aligned} P_p(t)=L{\textsf {Q}}\omega _1 \cos (\omega _1 t). \end{aligned}$$

The quantum behavior of the system in this case is illustrated in detail in the text.
